# Campylobacter jejuni Bacteremia in an Immunocompromised Host: A Rare Case Report

**DOI:** 10.7759/cureus.106793

**Published:** 2026-04-10

**Authors:** Vismay Patel, Hassan Waheed, Rubba S Khan, Maryam Aslani, Jack Boghossian, Rehan Shah, Hammad Choudhry

**Affiliations:** 1 Internal Medicine, Hudson Regional Health (HRH) Bayonne University Hospital, Bayonne, USA; 2 Infectious Diseases, St. Michael Medical Center, Newark, USA; 3 Internal Medicine, Hudson Regional Health, Bayonne, USA

**Keywords:** abdominal pain, bacteremia, blood culture, camphylobacter jejuni, colitis

## Abstract

*Campylobacter jejuni* is a common cause of bacterial gastroenteritis and typically produces a self-limited illness in immunocompetent hosts. Bacteremia is rare and is usually described in patients with immunosuppression or significant comorbidities, with risk for severe systemic complications if diagnosis is delayed. We report a case of *Campylobacter jejuni *bacteremia in a 41-year-old man with type 2 diabetes mellitus, coronary artery disease, hypertension, hyperlipidemia, and obstructive sleep apnea who presented with fever, chills, and profuse watery diarrhea after consuming convenience-store food. Blood cultures obtained at an outside facility later grew *C. jejuni*. By the time of admission, gastrointestinal symptoms had been resolved, and he was hemodynamically stable. Laboratory evaluation showed normal leukocyte count and mild transaminitis, and computed tomography demonstrated ileocecal enterocolitis. He was treated with azithromycin, and repeat blood cultures were negative; he achieved complete clinical recovery. This case emphasizes the importance of reviewing pending blood culture results and initiating timely macrolide therapy to prevent systemic complications. This case is notable for delayed bacteremia despite resolution of gastrointestinal symptoms and highlights the importance of reviewing pending blood culture results.

## Introduction

*Campylobacter jejuni* is one of the most common bacterial causes of gastroenteritis worldwide, accounting for an estimated 5-14% of diarrheal illnesses and approximately 1.5 million infections annually in the United States [1,2]. Transmission typically occurs through ingestion of contaminated food or water, most commonly undercooked poultry, unpasteurized dairy products, or cross-contaminated ready-to-eat foods [1-3]. In immunocompetent individuals, infection is usually self-limited; however, extraintestinal manifestations may occur, particularly in patients with underlying comorbidities or impaired host defenses [4,5].

Bacteremia due to *C. jejuni* is rare, reported in fewer than 1% of *Campylobacter* infections, but associated with significantly increased morbidity and mortality [4,6]. Population-based studies estimate the incidence of *Campylobacter* bacteremia at approximately 0.2-0.4 cases per 100,000 persons per year, with higher rates among the elderly and patients with multiple metabolic comorbidities [4-6]. Patients with diabetes mellitus, cardiovascular disease, malignancy, chronic liver disease, or other immunosuppressive conditions are disproportionately affected [5,6]. Reported mortality rates range from 5% to 15%, increasing substantially in patients with severe comorbidities or delayed initiation of appropriate antimicrobial therapy [4,6].

Hematogenous dissemination may result in severe systemic complications, including sepsis, infective endocarditis, mycotic aneurysm, and vascular prosthesis infection, particularly with invasive species such as *C. fetus* [7,8]. Importantly, bacteremia may develop even after gastrointestinal symptoms have been resolved, which can delay diagnosis and treatment. Prompt recognition and initiation of effective antimicrobial therapy, most commonly macrolides, are therefore essential to prevent progression to life-threatening disease [2,9]. This case is notable for delayed bacteremia despite resolution of gastrointestinal symptoms and highlights the importance of reviewing pending blood culture results.

## Case presentation

A 41-year-old man with type 2 diabetes mellitus, coronary artery disease (status post coronary artery bypass grafting and prior stent placement), hypertension, hyperlipidemia, and obstructive sleep apnea on continuous positive airway pressure (CPAP) presented with fever, chills, and profuse watery diarrhea occurring approximately 10-15 times per day after eating convenience-store food. He denied hematochezia, melena, nausea, vomiting, recent travel, sick contacts, or antibiotic use prior to symptom onset. At an outside facility, blood cultures were obtained, and the patient was discharged on empiric amoxicillin-clavulanate for presumed bacterial gastroenteritis, although *Campylobacter* species are often resistant to beta-lactams. He was subsequently contacted and advised to return when blood cultures grew *Campylobacter jejuni*, prompting presentation to our emergency department. 

On arrival, the patient was hemodynamically stable and afebrile. Vital signs were within normal limits. Physical examination revealed a soft, nondistended abdomen without tenderness, rebound, or guarding. Bowel sounds were present, and there were no signs of peritoneal irritation. At that time, his diarrhea had largely resolved, and he reported improvement in systemic symptoms. 

Differential diagnoses included inflammatory bowel disease, ischemic colitis, and other infectious etiologies; however, clinical and microbiologic findings supported infectious enterocolitis.

Laboratory studies revealed a normal leukocyte count with neutrophil predominance, which is consistent with systemic bacterial infection, while mild transaminitis may reflect systemic inflammatory response or enteric infection, and mild transaminitis. Hemoglobin, platelet count, renal function, and electrolytes were within normal limits. Mild respiratory acidosis was likely related to underlying obstructive sleep apnea and was clinically incidental. Serum lactate was normal. Viral hepatitis panel and HIV screening were negative, and coagulation parameters were within normal range (Table 1).

**Table 1:** Laboratory evaluation revealed a normal leukocyte count with neutrophil predominance and mild transaminitis. WBC: white blood cell count; BUN: blood urea nitrogen; eGFR: estimated glomerular filtration rate; AST: aspartate aminotransferase; ALT: alanine aminotransferase; CO₂: carbon dioxide; pCO₂: partial pressure of carbon dioxide; HCO₃: bicarbonate; PT: prothrombin time; INR: international normalized ratio; aPTT: activated partial thromboplastin time; HIV: human immunodeficiency virus.

Test	Result	Reference Range	Interpretation
Complete Blood Count			
WBC	8.4 ×10³/µL	4.0–11.0	Normal
Hemoglobin	15.9 g/dL	13.5–17.5	Normal
Hematocrit	46.3 %	41–53	Normal
Platelets	241 ×10³/µL	150–400	Normal
Neutrophils	80.4 %	40–70	↑
Lymphocytes	9.5 %	20–40	↓
Basic Metabolic Panel			
Sodium	140 mmol/L	135–145	Normal
Potassium	4.3 mmol/L	3.5–5.0	Normal
Chloride	103 mmol/L	98–107	Normal
Bicarbonate (CO₂)	25 mmol/L	22–29	Normal
BUN	20 mg/dL	7–20	Normal
Creatinine	1.3 mg/dL	0.6–1.3	Upper-normal
eGFR	71 mL/min/1.73 m²	>60	Normal
Glucose (random)	87 mg/dL	70–140	Normal
Calcium	9.7 mg/dL	8.6–10.2	Normal
Magnesium	2.3 mg/dL	1.7–2.2	↑
Phosphorus	3.9 mg/dL	2.5–4.5	Normal
Liver Function Tests			
AST	60 U/L	10–40	↑
ALT	64 U/L	7–56	↑
Alkaline Phosphatase	66 U/L	40–129	Normal
Total Bilirubin	1.2 mg/dL	0.2–1.2	Upper-normal
Albumin	4.8 g/dL	3.5–5.0	Normal
Total Protein	8.1 g/dL	6.0–8.3	Normal
Venous Blood Gas			
pH	7.32	7.35–7.45	Mild acidosis
pCO₂	57 mmHg	35–45	↑
HCO₃⁻	29.4 mmol/L	22–26	↑
Lactate	1.4 mmol/L	0.5–2.0	Normal
Coagulation			
PT	12.9 sec	11–13.5	Normal
INR	1.16	0.8–1.2	Normal
aPTT	34.9 sec	25–35	Normal
Infectious Serologies			
Hepatitis A IgM	Negative	—	—
Hepatitis B surface antigen	Negative	—	—
Hepatitis B core IgM	Negative	—	—
Hepatitis C antibody	Negative	—	—
HIV 1/2 Ag/Ab (4th gen)	Negative	—	—

**Figure 1:**
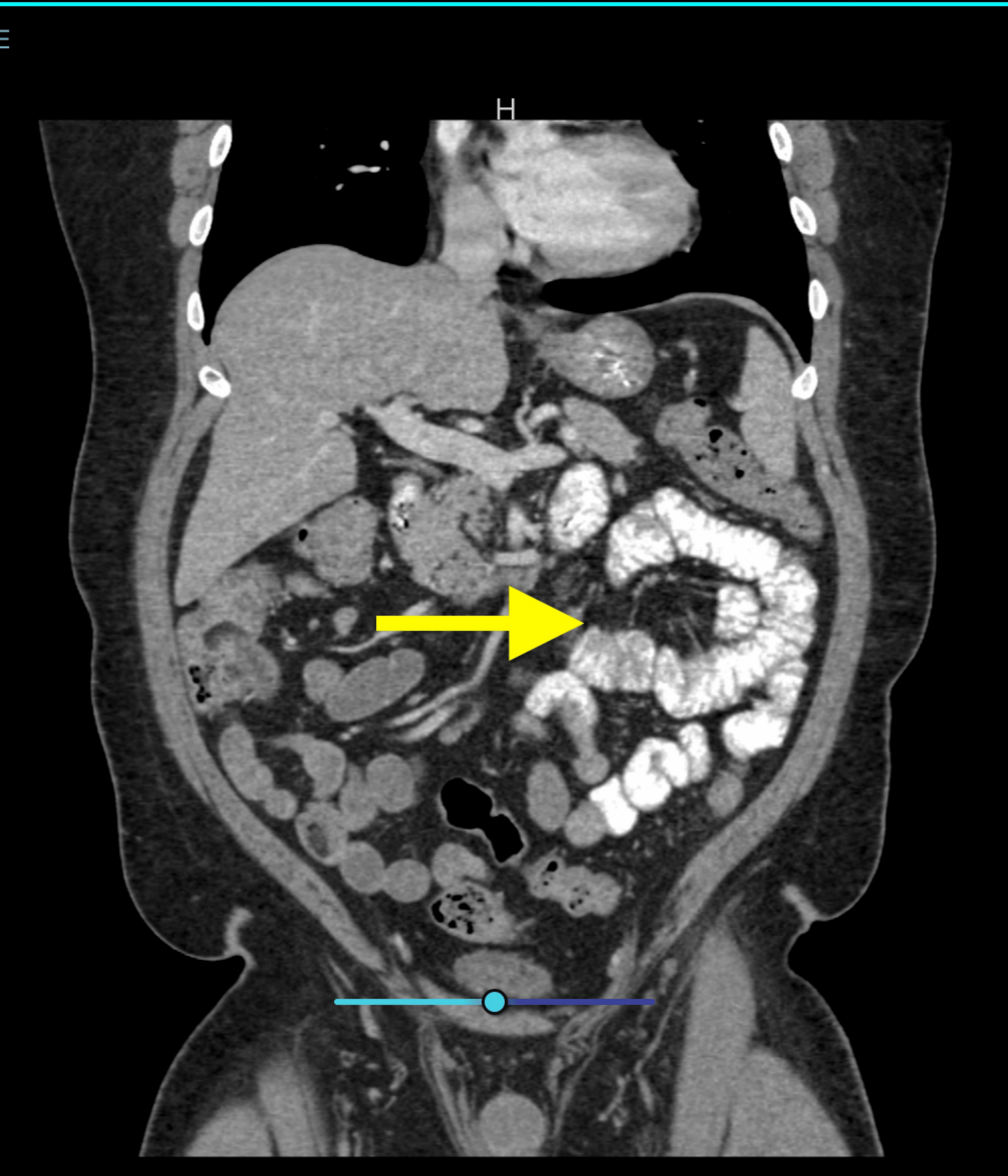
Computed tomography of the abdomen from the referring hospital demonstrated mural thickening and mucosal hyperenhancement of the terminal ileum and right colon with mild surrounding fat stranding, findings consistent with infectious enterocolitis.

Computed tomography of the abdomen from the referring hospital demonstrated mural thickening and mucosal hyperenhancement of the terminal ileum and right colon with mild surrounding fat stranding; these findings are nonspecific but supportive of infectious enterocolitis and primarily served a confirmatory role (Figure 1).

During hospitalization, intravenous fluids and azithromycin 500 mg once daily for five days were initiated as first-line therapy given increasing fluoroquinolone resistance [2-4]. An infectious disease consultation was obtained, and the patient was monitored with repeat blood cultures, which were negative prior to discharge. Stool studies were not performed as symptoms had resolved and the diagnosis was established via blood cultures. The patient remained clinically stable, repeat blood cultures were negative, and he was discharged with complete resolution of symptoms.

## Discussion

Although *Campylobacter jejuni* most often causes self-limited gastroenteritis, bacteremia can occur in patients with comorbidities or those who are immunosuppressed [1-4]. Our patient, though not classically immunosuppressed, had multiple metabolic comorbidities, such as diabetes and vascular disease, that may impair immune function and increase susceptibility to invasive infection, which likely predisposed him to systemic invasion.

Beyond *C. jejuni*, several *Campylobacter* species have been implicated in bloodstream infections. *C. coli*, *C. fetus*, and *C. upsaliensis* are among the most frequently identified species associated with bacteremia, particularly among patients with multiple metabolic comorbidities or elderly patients [1,2,5]. Observational studies indicate that while *C. jejuni* accounts for the majority of isolates; other species remain clinically significant pathogens, especially in high-risk hosts [5]. *C. fetus* demonstrates a strong predilection for systemic spread due to virulence factors that confer serum resistance and endothelial adherence, predisposing to endovascular infection and prosthetic material involvement [6]. Reports of vascular prosthesis infections and endocarditis reinforce its invasive potential [6].

Bacteremia may present after gastrointestinal symptoms have already improved, which can delay recognition. In this case, diarrhea had resolved by the time of admission, yet blood cultures flagged positive, emphasizing the importance of reviewing pending culture results from prior encounters to avoid missed or delayed treatment. Population-based analyses show that many patients with *Campylobacter bacteremia* no longer exhibit gastrointestinal symptoms at diagnosis [4,5].

It remains uncertain whether the apparent increase in reported *Campylobacter bacteremia* reflects a true rise in incidence or improved diagnostic capabilities. Enhanced blood culture systems and modern identification methods have led to more frequent detection of bloodstream infections caused by *Campylobacter* species, including previously underrecognized organisms [5,7].

Once in the bloodstream, *C. jejuni* and other species can cause serious systemic infections, including sepsis, endocarditis, and vascular complications if not treated appropriately [4,8,6]. Radiologic findings of mural thickening in the ileocecal region are nonspecific but supportive of infectious colitis in the appropriate clinical context.

Macrolides, especially azithromycin, remain first-line therapy given their effectiveness, since local resistance data were not available; however, increasing fluoroquinolone resistance has been widely reported [9-12]. Timely initiation of appropriate antimicrobial therapy correlates strongly with improved outcomes, whereas delays are associated with higher morbidity and mortality. Early antimicrobial treatment prevents progression to severe systemic complications. Our patient’s recovery illustrates the importance of prompt recognition and therapy.

## Conclusions

This case demonstrates that *Campylobacter jejuni* bacteremia, though rare, should be considered in patients with significant co-morbidities and recent diarrheal illness. Blood cultures remain critical for diagnosis, especially when symptoms improve, but risk factors persist. Early recognition and treatment with macrolides can prevent progression to severe infection and improve outcomes, and ongoing surveillance will clarify whether rising case numbers reflect true epidemiologic change or enhanced detection. While limited to a single case, this highlights the importance of reviewing pending microbiological results to avoid missed diagnoses.

## References

[1] Barral M, Boudiaf M, Dohan A (2015). MDCT of acute colitis in adults: an update in current imaging features. Diagn Interv Imaging.

[2] (2025). Centers for Disease Control and Prevention. Clinical overview of Campylobacter. https://www.cdc.gov/campylobacter/hcp/clinical-overview/index.html.

[3] Shane AL, Mody RK, Crump JA (2017). 2017 Infectious Diseases Society of America clinical practice guidelines for the diagnosis and management of infectious diarrhea. Clin Infect Dis.

[4] Tribble DR (2017). Antibiotic therapy for acute watery diarrhea and dysentery. Mil Med.

[5] Pacanowski J, Lalande V, Lacombe K (2008). Campylobacter bacteremia: clinical features and factors associated with fatal outcome. Clin Infect Dis.

[6] Fernández-Cruz A, Muñoz P, Mohedano R (2010). Campylobacter bacteremia: clinical characteristics, incidence, and outcome over 23 years. Medicine (Baltimore).

[7] Sunnerhagen T, Grenthe R, Kampmann C (2024). Campylobacter infections with and without bacteremia: a comparative retrospective population-based study. Open Forum Infect Dis.

[8] Graham A, Hawkins L, Balasegaram S (2024). A decade of Campylobacter and Campylobacter bacteraemias in a district general hospital and the surrounding London and South East region, England. J Infect.

[9] Moreira GS, Feijóo NA, Tinoco-da-Silva IB (2024). Splenic embolism in infective endocarditis: a systematic review of the literature with an emphasis on radiological and histopathological diagnoses. Trop Med Infect Dis.

[10] Nielsen H, Hansen KK, Gradel KO (2010). Bacteraemia as a result of Campylobacter species: a population-based study of epidemiology and clinical risk factors. Clin Microbiol Infect.

[11] Otsuka Y, Hagiya H, Takahashi M (2023). Clinical characteristics of Campylobacter bacteremia: a multicenter retrospective study. Sci Rep.

[12] Moffatt CR, Kennedy KJ, O'Neill B (2021). Bacteraemia, antimicrobial susceptibility and treatment among Campylobacter-associated hospitalisations in the Australian Capital Territory: a review. BMC Infect Dis.

